# 70 Burn Injury is Not a Risk Factor for Long-term Opioid Use

**DOI:** 10.1093/jbcr/irad045.044

**Published:** 2023-05-15

**Authors:** David Keeven, Jonathan Vacek, Matthew Bozeman, Keith Miller, Nicholas Nash, Matthew Benns, Samuel Pera, Jamie Coleman, William Risinger, Glen Franklin

**Affiliations:** University of Louisville, Louisville, Kentucky; University of Louisville, Louisville, Kentucky; University of Louisville Hospital Burn Center/UofL Health, Louisville, Kentucky; University of Louisville, Louisville, Kentucky; University of Louisville, Louisville, Kentucky; University of Louisville, Louisville, Kentucky; University of Louisville, Louisville, Kentucky; University of Louisville, Louisville, Kentucky; University of Louisville, Louisville, Kentucky; University of Louisville, Louisville, Kentucky; University of Louisville, Louisville, Kentucky; University of Louisville, Louisville, Kentucky; University of Louisville Hospital Burn Center/UofL Health, Louisville, Kentucky; University of Louisville, Louisville, Kentucky; University of Louisville, Louisville, Kentucky; University of Louisville, Louisville, Kentucky; University of Louisville, Louisville, Kentucky; University of Louisville, Louisville, Kentucky; University of Louisville, Louisville, Kentucky; University of Louisville, Louisville, Kentucky; University of Louisville, Louisville, Kentucky; University of Louisville, Louisville, Kentucky; University of Louisville Hospital Burn Center/UofL Health, Louisville, Kentucky; University of Louisville, Louisville, Kentucky; University of Louisville, Louisville, Kentucky; University of Louisville, Louisville, Kentucky; University of Louisville, Louisville, Kentucky; University of Louisville, Louisville, Kentucky; University of Louisville, Louisville, Kentucky; University of Louisville, Louisville, Kentucky; University of Louisville, Louisville, Kentucky; University of Louisville, Louisville, Kentucky; University of Louisville Hospital Burn Center/UofL Health, Louisville, Kentucky; University of Louisville, Louisville, Kentucky; University of Louisville, Louisville, Kentucky; University of Louisville, Louisville, Kentucky; University of Louisville, Louisville, Kentucky; University of Louisville, Louisville, Kentucky; University of Louisville, Louisville, Kentucky; University of Louisville, Louisville, Kentucky; University of Louisville, Louisville, Kentucky; University of Louisville, Louisville, Kentucky; University of Louisville Hospital Burn Center/UofL Health, Louisville, Kentucky; University of Louisville, Louisville, Kentucky; University of Louisville, Louisville, Kentucky; University of Louisville, Louisville, Kentucky; University of Louisville, Louisville, Kentucky; University of Louisville, Louisville, Kentucky; University of Louisville, Louisville, Kentucky; University of Louisville, Louisville, Kentucky; University of Louisville, Louisville, Kentucky; University of Louisville, Louisville, Kentucky; University of Louisville Hospital Burn Center/UofL Health, Louisville, Kentucky; University of Louisville, Louisville, Kentucky; University of Louisville, Louisville, Kentucky; University of Louisville, Louisville, Kentucky; University of Louisville, Louisville, Kentucky; University of Louisville, Louisville, Kentucky; University of Louisville, Louisville, Kentucky; University of Louisville, Louisville, Kentucky; University of Louisville, Louisville, Kentucky; University of Louisville, Louisville, Kentucky; University of Louisville Hospital Burn Center/UofL Health, Louisville, Kentucky; University of Louisville, Louisville, Kentucky; University of Louisville, Louisville, Kentucky; University of Louisville, Louisville, Kentucky; University of Louisville, Louisville, Kentucky; University of Louisville, Louisville, Kentucky; University of Louisville, Louisville, Kentucky; University of Louisville, Louisville, Kentucky; University of Louisville, Louisville, Kentucky; University of Louisville, Louisville, Kentucky; University of Louisville Hospital Burn Center/UofL Health, Louisville, Kentucky; University of Louisville, Louisville, Kentucky; University of Louisville, Louisville, Kentucky; University of Louisville, Louisville, Kentucky; University of Louisville, Louisville, Kentucky; University of Louisville, Louisville, Kentucky; University of Louisville, Louisville, Kentucky; University of Louisville, Louisville, Kentucky; University of Louisville, Louisville, Kentucky; University of Louisville, Louisville, Kentucky; University of Louisville Hospital Burn Center/UofL Health, Louisville, Kentucky; University of Louisville, Louisville, Kentucky; University of Louisville, Louisville, Kentucky; University of Louisville, Louisville, Kentucky; University of Louisville, Louisville, Kentucky; University of Louisville, Louisville, Kentucky; University of Louisville, Louisville, Kentucky; University of Louisville, Louisville, Kentucky; University of Louisville, Louisville, Kentucky; University of Louisville, Louisville, Kentucky; University of Louisville Hospital Burn Center/UofL Health, Louisville, Kentucky; University of Louisville, Louisville, Kentucky; University of Louisville, Louisville, Kentucky; University of Louisville, Louisville, Kentucky; University of Louisville, Louisville, Kentucky; University of Louisville, Louisville, Kentucky; University of Louisville, Louisville, Kentucky; University of Louisville, Louisville, Kentucky

## Abstract

**Introduction:**

The United States continues to suffer from a serious epidemic of opioid use. Exposure to opioids is a known risk factor for long-term use and dependence. This is of particular importance to burn care, as opioids are frequently essential to manage the pain associated with burn injuries. The purpose of this study was to characterize opioid use among burn patients after hospitalization and to identify any risk factors for long-term dependence.

**Methods:**

All patients admitted to a burn center during a single year period (2/1/2020-2/1/21) were examined. Patients who died were excluded. A controlled substance reporting system was utilized to determine opioid use over a time period from 6 months prior to injury and up to 12 months post-hospital discharge. Reporting to this database is mandated by law and therefore includes all prescriptions regardless of prescriber, insurance, location, etc. Duration of opioid use was recorded for all patients in the study. Long-term use was defined as having an active opioid prescription at 12 months post-hospital discharge. Patient demographic information, burn injury characteristics, operative interventions, and hospital course were examined to determine risk factors for long-term opioid use following burn injury.

**Results:**

A total of 185 patients were included in the study. Patients were predominantly male (76.2%) and had an average age of 47.5 years old. Patients had an average TBSA burn of 9.5% and a length of stay of 6.7 days. A total of 54.7% of patients received an opioid prescription at discharge. Only 17 patients (9.7%) had persistent opioid use at 1 year. The only independent risk factor identified for long term opioid use was pre-injury use (p< 0.001). Among the 17 patients still on opioids at 1 year, 16 had filled 3 or more opioid prescriptions in the 6 months prior to injury. The solitary patient without a pre-existing opioid history had multiple readmissions and delayed operative interventions after their initial hospital course.

**Conclusions:**

A majority of burn patients received opioids for pain control following hospital discharge. However, the duration of therapy was generally short among opioid naïve patients. Burn injury was not associated with long term opioid use among patients in the study.

**Applicability of Research to Practice:**

When used judiciously and with caution, opioids can be safely prescribed to burn patients without fear of long-term opioid dependence.

